# Volatile Anaesthesia versus Total Intravenous Anaesthesia for Cardiac Surgery—A Narrative Review

**DOI:** 10.3390/jcm11206031

**Published:** 2022-10-13

**Authors:** Mihai Ștefan, Cornelia Predoi, Raluca Goicea, Daniela Filipescu

**Affiliations:** 1 Department of Anaesthesiology and Intensive Care, “Prof Dr CC Iliescu” Emergency Institute for Cardiovascular Diseases, 022322 Bucharest, Romania; 2 Discipline of Anaesthesiology and Intensive Care, “Carol Davila” University of Medicine and Pharmacy, 050474 Bucharest, Romania

**Keywords:** cardiac anaesthesia, cardiac surgery, total intravenous anaesthesia, volatile anaesthetics, anaesthetic preconditioning, myocardial ischemia

## Abstract

Recent research has contested the previously accepted paradigm that volatile anaesthetics improve outcomes in cardiac surgery patients when compared to intravenous anaesthesia. In this review we summarise the mechanisms of myocardial ischaemia/reperfusion injury and cardioprotection in cardiac surgery. In addition, we make a comprehensive analysis of evidence comparing outcomes in patients undergoing cardiac surgery under volatile or intravenous anaesthesia, in terms of mortality and morbidity (cardiac, neurological, renal, pulmonary).

## 1. Introduction

Cardiac surgery presents unique challenges to the anaesthesiologists as they are responsible for providing amnesia, analgesia, muscle relaxation and maintaining organ functions in the context of the pathophysiologic changes induced by the cardiopulmonary bypass (CPB). The use of CPB results in pharmacokinetic and pharmacodynamic alterations that impact the serum and tissue concentrations of intravenous (iv.) and volatile anaesthetics (VA), as well as their efficacy [1]. In addition, monitors of anaesthetic depth to help anaesthesia management are often unreliable or directly impacted by the CPB. After adult cardiac surgery, postoperative complications such as myocardial infarction (MI), low cardiac output syndrome (LCOS), stroke, delirium, postoperative cognitive dysfunction (POCD), hypoxemia, pneumonia, acute respiratory distress syndrome (ARDS), acute kidney injury (AKI) and the need of renal replacement therapy (RRT), prolong the intensive care unit (ICU) and in-hospital length of stay (LOS), and increase the mortality rate [2]. There are several interventions which could improve perioperative outcome, one of which is the adequate, safe administration of anaesthesia [3].

However, whether one anaesthetic regimen is superior to the other in cardiac surgery is debated. As several clinical trials conducted in patients undergoing coronary artery bypass grafting (CABG) surgery suggested relevant cardioprotection, the 2011 American College of Cardiology Foundation (ACCF)/American Heart Association (AHA) guideline gave a class IIa level A recommendation for the use of VA with the aim of reducing the risk of perioperative myocardial ischaemia and infarction [4]. Furthermore, the 2017 European Society of Cardiac Thoracic Surgery (EACTS) guideline, acknowledging that halogenated anaesthetics (isoflurane, desflurane, sevoflurane) versus (vs.) total iv. anaesthesia (TIVA) resulted in additional organ protection and improvements in clinically relevant endpoints after CABG, including a reduction in mortality and perioperative MI rates in a couple of studies and meta-analyses, gave a class I B recommendation for the use of an anaesthetic regimen which included VA in CABG patients [5]. In contrast, as new evidence published in 2019 did not support the favourable effects of VA [6], the EACTS/European Association of Cardiothoracic Anaesthesiology (EACTA)/European Board of Cardiovascular Perfusion (EBCP) guidelines on CPB in adult cardiac surgery gave only a class IIa B recommendation for the use of VA during CPB [7].

Interestingly, a recent systematic review on anaesthetic management during CPB stated that the use of VA predominates in North America, and that iv. agents are more common in Europe [1]. In a survey of North American members of the Society of Cardiovascular Anesthesiologists, most respondents indicated that they prefer VA for maintenance of anaesthesia, that anaesthetic selection impacts patient outcomes, and that VA have organ-protective properties [8]. However, the members’ motivation for preferring these agents reflected more practical considerations, such as ease of use, effectiveness, and institutional practice, including the fact that the perfusionist delivered the anaesthetic during the CPB. In contrast, a European survey showed that only 36% centres used VA during CPB [9]. This difference compared to North American practice, likely arises from an old European Council directive prohibiting the attachment of an anaesthetic vaporizer to a CPB machine [10].

This review provides a description of the mechanisms of cardiac dysfunction after CPB, addresses the potential of anaesthetics in preventing postoperative complications, and summarizes the current evidence on the influence of the anaesthetic regimen (VA or iv.) on outcomes after cardiac surgery.

## 2. Pathophysiology of Myocardial Dysfunction and Mechanisms of Cardioprotection in Cardiac Surgery

### 2.1. Impact of Cardiac Surgery with CPB on Cardiac Function

During cardiac surgery with CPB, after aortic cross-clamping, the heart is isolated from the circulation, and this inevitably induces myocardial ischaemia. After a period of myocardial ischaemia of variable duration, further reperfusion may lead to additional injury beyond that generated by the ischemia and may manifest as arrhythmias, reversible contractile dysfunction (myocardial stunning), endothelial dysfunction and ultimately irreversible injury with myocardial cell death [1,11]. The magnitude of myocardial ischaemia is highly variable and depends upon the severity of the underlying disease, the duration of aortic cross-clamping, and the quality of myocardial protection during CPB [12]. Myocardial injury can result in delayed recovery, organ failure, increased hospital LOS, and mortality.

**Figure 1:**
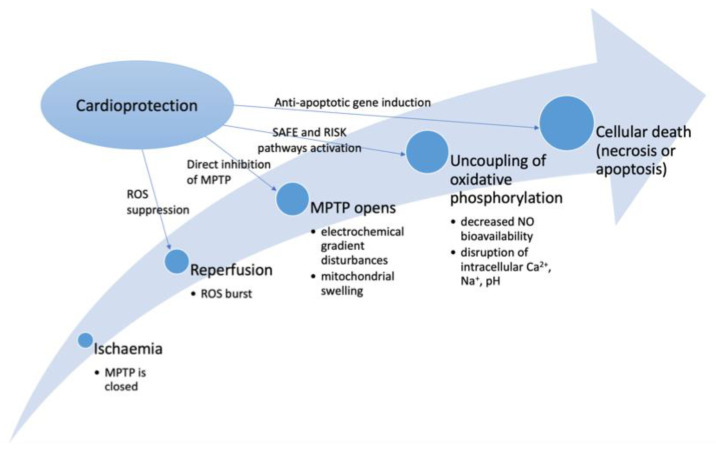
Summary of mechanisms of ischaemia reperfusion and cardioprotection. Legend: ROS—reactive oxygen species, MPTP—mitochondrial permeability transition pore, SAFE—survivor activating factor enhancement, RISK—reperfusion injury salvage kinase, NO—nitrous oxide.

Central in the pathogenesis of ischaemic myocardial injury is the depletion of high-energy phosphates and the disturbance of normal intracellular calcium (Ca^2+^) homeostasis [11,13]. The magnitude of reperfusion injury is dependent on the extent of damage to the mitochondrion, which is in turn proportional to the degree of opening of the mitochondrial permeability transition pore (MPTP), ultimately leading to cardiomyocyte death and subsequent irreversible myocardial injury [14]. During ischaemia, the MPTP remains closed, maintaining mitochondrial integrity. Upon reperfusion, the tissue re-oxygenation triggers the release of reactive oxygen species (ROS) and the MPTP opens, leading to the disturbance of electrochemical gradients over the mitochondrial membrane, swelling of the mitochondrial intermembrane space and disruption of the supramolecular complex containing the proton pump, adenosine triphosphate (ATP) synthase, the adenine nucleotide transporter and mitochondrial creatine kinase (CK), which uncouples oxidative phosphorylation, decreases nitric oxide (NO) bioavailability, and disrupts intracellular distribution of Ca^2+^, sodium (Na^+^), potassium (K^+^) and hydrogen ions [13,14]. If the MPTP opening is minimal, full functional recovery of the mitochondrion may occur. Depending on the extent of damage and the amount of MPTP opening either recovery or apoptosis or necrosis of cell will occur leading to irreversible damage to the myocardium [11]. Maintaining the mitochondrial chain function is crucial to preserve adenosine triphosphate (ATP) production and to prevent detrimental oxidative damage (Figure 1).

### 2.2. Cardioprotection

The mechanisms involved in cardioprotection account for decreased cytosolic and mitochondrial Ca^2+^ loading with the final objective of maintaining intracellular homeostasis through the preservation of the mitochondrion and its normal function [13,15].

**Figure 2:**
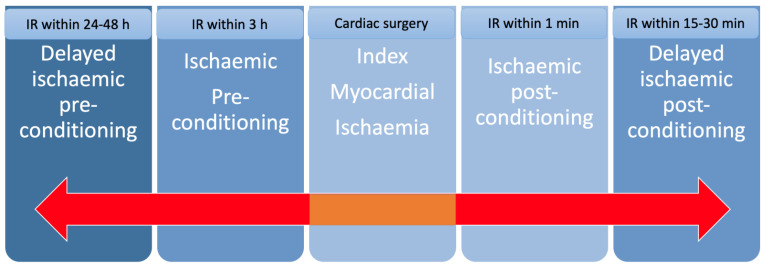
Types of ischaemic conditioning and relation to surgery according to ref. [17]. Legend: IR—ischaemia reperfusion.

Cardioprotection against the deleterious effects of myocardial ischaemia-reperfusion (IR) injury is an adaptive response to increase myocardial resistance to irreversible IR injury. This can be elicited by applying brief cycles of ischaemia and reperfusion directly to the heart [16]. Depending on the timing of these IR cycles they are described as ischaemic pre-conditioning (IPC) (within 3 h of the index myocardial ischaemia), delayed IPC (24–48 h prior the myocardial index ischaemia), ischaemic post-conditioning (within 1 min of reperfusion following the index myocardial ischaemia) and delayed ischaemic post-conditioning (15–30 min after the onset of myocardial reperfusion following the index myocardial ischaemia) (Figure 2) [16].

The concept of “conditioning” refers to a combination of pre- and postconditioning, which exerts synergistic interactions. After the first stimulus, a second window of protection occurs hours later, being a result of activated transcriptional factors which alter gene expression [18].

Cardioprotection can also be induced by pharmacological agents, including anaesthetic agents, or by applying brief cycles of ischaemia and reperfusion to an organ or tissue (such as the arm or leg) away from the heart known as “remote ischaemic preconditioning” (RIPC) [13,16].

### 2.3. Anaesthetic Cardioprotection

Similar to ischaemic conditioning, anaesthetic conditioning protects the heart and other organs against IR injury through the survivor activating factor enhancement (SAFE) and reperfusion injury salvage kinase (RISK) pathways, which, subsequently, through mitochondrial-dependent and non-mitochondrial-dependent (nuclear or other) signalling pathways decrease MPTP opening, increase mitochondrial K_ATP_ channel opening, activate adenosine receptors, and inhibit Na+/K+ pump which attenuates IR injury [13,14]. Activation of the endogenous protection programme in the heart results in long term protective/resistance against IR [18]. Other organs may also benefit from anaesthetic conditioning protective effects.

VA have both pre- and postconditioning effects and the specific cardioprotective properties are independent of the hypnotic properties of the gases [13]. The exposure of cardiomyocytes to VA before, during, and after aortic cross-clamping for coronary bypass anastomosis mimics ischaemic conditioning and triggers multiple signalling pathways to “prepare” the cell for the attendant hypoxia, rendering it more resistant to hypoxic stress damage, a process which is called anaesthetic conditioning [19,20].

VA activate the intracellular signal cascade via G-protein-coupled receptors, which results in less mitochondrial Ca^2+^ and more mitochondrial K^+^, less ROS, and a direct inhibition of the MPTPs, as well as altering the gene expression in the cardiomyocyte, leading to a transcription of anti-apoptotic genes, expression of some of the key enzymes involved in VA cardioprotection and opening the “second window” of protection [18,21].

Preconditioning with VA (isoflurane-before CPB, and sevoflurane before and after CPB) also prevents oxidative and nitrosative stress during CABG surgery through overexpression of antioxidant enzymes [21].

An important aspect of anaesthetic cardioprotection is the time point of application of VA in relation to the IR event. In one study, a protective effect as evidenced by a lower postoperative cardiac troponin (cTn) T release and a better myocardial performance index, was observed only with the intermittent administration of VA [17]. VA might induced a protective signal in the myocardial cells in defined windows only, suggesting that the amount of VA administered is not critical [22]. Other authors suggest that the administration of VA throughout the entire procedure results in a more pronounced protective effect than when administered intermittently [23,24] or only before or after CPB [25]. Moreover, cardioprotective effects seemed to be related to the amount of VA administered as laboratory investigations reported 1.0 MAC of VA associated with beneficial effect to cardiac injury and that lower concentrations or more than 1.5 MAC did not result in further protective effect [26].

The above discussion points to the fact that the optimal protocol of VA administration for cardioprotection is unknown. On the other hand, molecular interactions interfering with VA-induced cardioprotection are frequent. Genetic susceptibility, advanced age, male sex, obesity, hyperglycaemia, hyperlipidaemia, hypertension and diabetes mellitus diminish its effectiveness similar to several non-VA agents (propofol, sulfonylureas, betablockers) [27,28]. In contrast, other factors, such as the presence of angina, the concomitant administration of P2Y12 receptor antagonists or statins and intralipid, most notably for the latter when administered before aortic cross-clamping, promote cardioprotection [28,29,30].

Iv. anaesthetics are also reported to have cardioprotective effects related to anti-inflammatory, immuno-modulatory and antioxidant properties [13,31]. The scavenging of ROS or the regulation of Ca+ overload during reperfusion may confer pre- or postconditioning-like effects of propofol [18]. In experimental studies propofol improved cardiac mechanics in IR and decreased the MI size, but the cardioprotective role remains controversial as it does not elicit the signaling pathways of pharmacological conditioning. On the other hand, propofol can impede the mitochondrial respiratory chain and inhibit the protective signalling pathway initiated by VA by scavenging the required “signalling” ROS and therefore activating the MPTP channels and inhibition of K_ATP_ channels [13,14,18]. Preservation of mitochondrial respiration and cardioprotection against IR resulting in decreased myocardial infarction size has been recently confirmed for sevoflurane but not for propofol anaesthesia [32].

Noteworthy, propofol was shown to be a pre-emptive intraoperative cardioprotective drug for patients with diabetes mellitus under conditions of normothermic bypass and blood cardioplegic arrest, resulting in decreased episodes of LCOS and heart failure events following cardiac surgery [33]. Patients who received propofol during CPB had higher levels of mitochondrial protectants and higher levels of oxidative stress at reperfusion compared with patients who received isoflurane, suggesting a pro-oxidant mechanism of cardioprotection mediated by propofol. On subgroup analysis, the clinical benefit was consistent in diabetic patients and not apparent in non-diabetic patients, suggesting that propofol may have its own cardioprotective properties that are only clinically apparent in certain populations.

Anti-inflammatory effects may also contribute to propofol-related cardioprotection [34]. Moreover, a previous RCT reported that compared with VA, TIVA could control stress and hemodynamic response in patients undergoing CABG surgery, contributing to a cardioprotective effect [35].

Interestingly, the combination of isoflurane preconditioning and propofol postconditioning effects resulted in decreased postoperative isoenzyme MB of CK and cTn I release and facilitated postoperative myocardial functional recovery compared to the control group anesthetized with fentanyl and midazolam, suggesting a potential synergism in modulating the IR injury after CPB [36].

The diverging properties of different anaesthetic regimens on IR injury highlight the challenges in translating cardioprotective strategies to the clinical setting [13]. Consequently, clinically meaningful cardioprotective effects of anaesthetics remain controversial.

## 3. Clinical Translation of Anaesthetic Cardioprotection

### 3.1. Biomarker Alterations

The cardioprotective potential of various anaesthetics used during cardiac surgery may be documented by a reduction in biomarkers of cardiac injury. Several randomized controlled trials (RCTs) and relevant meta-analyses suggested that VA (in particular sevoflurane and desflurane), mimicking IPC, might reduce perioperative myocardial damage in cardiac surgery, quantified by the level of cTn [25,37]. A meta-analysis which included 30 RCTs and 2578 patients showed significantly lower postoperative peak serum levels of cTn I in patients receiving a VA regimen compared with an iv. anaesthetic regimen [0.995 mg/L; standard mean difference, 95% confidence interval (CI), 1.316 to 0.673; *p* < 0.001] [25]. VA reduced postoperative cTn I concentrations by approximately 8% in on-pump surgery, but a protective effect was not evident in off-pump coronary artery bypass (OPCAB) surgery, which still exposes patients to myocardial IR injury. The trial sequential analysis (TSA) indicated that the combined existing trials were of sufficient power and low heterogeneity to affirm that there is no need for further studies looking into surrogate markers of myocardial injury and VA protection in on-pump cardiac surgery but the results for OPCAB surgery were not conclusive [38].

Further studies confirmed the cardioprotective properties of VA compared to TIVA in terms of cardiac biomarker release [39,40]. A RCT performed in 868 patients undergoing CABG surgery with CPB found both cTn T (0.18 ng/mL vs. 0.57 ng/mL at 24 h, *p* < 0.001) and N-terminal pro-brain natriuretic peptide (NT- proBNP) (633 pg/mL vs. 878 pg/mL at 24 h, *p* < 0.001; 482 pg/mL vs. 1036 pg/mL at 48 h, *p* < 0.001) levels decreased in sevoflurane group compared to TIVA [40]. Significant reductions in cTn I levels were also found cardiac surgery patients who underwent RPIC in the isoflurane group compared with the propofol group [41].

Interestingly, others did not find differences in cTn T [42] or NT-proBNP levels [43] between VA and TIVA, pointing out to the importance of global cardioprotective measures applied and the protocol of VA administration.

Notably, in one old study, high-dose propofol administered while on CPB was associated with a cardioprotective effect quantified by the level of cTn I and cardiac index, in contrast to lower-dose propofol or isoflurane [44].

In OPCAB surgery, the benefits reported by some authors could be related to the use of sedation with sevoflurane in the postoperative period which enhanced the intraoperative cardioprotective properties [15,45]. This was confirmed in a further RCT where sevoflurane was associated with lowered cTn I levels, reduced need for inotropic support, better preserved renal function, and shorter ICU LOS compared with propofol or a combination of propofol and sevoflurane [46]. In contrast, when VA was used for anaesthesia and sedation in patients undergoing on-pump CABG, there were no differences in the levels of cardiac biomarkers compared to patient receiving propofol based TIVA [47].

No attempt was made in the above meta-analysis to compare different agents within VA group, because the pooled data were too sparse [25,38]. A noninferiority, single-centre RCT investigating patients receiving isoflurane vs. sevoflurane during CABG found no differences in the primary outcome of a composite of ICU LOS and mortality at 48 h and 30 days but did find a higher cTn level in the isoflurane versus sevoflurane group [48].

Although it was established that the use of a VA regimen during on-pump coronary artery surgery was associated with a lower post-operative cTn release compared with an iv. anaesthetic regimen, whether these findings result in improved clinically outcomes is currently controversial [2,38].

### 3.2. Mortality

The first larger clinical RCTs demonstrating benefits of VA compared to TIVA were published by the group of De Hert and colleagues in 2004 and 2009. They analysed 934 patients in total, and showed reduced ICU LOS and inotropic support for sevoflurane and desflurane, decreased ICU and in-hospital LOS for sevoflurane, and reduced 1-year mortality and in-hospital LOS for sevoflurane and desflurane, compared to midazolam or propofol based TIVA, propofol based TIVA and TIVA, respectively [20,23,42]. Notably, there were large differences in 1-year mortality between VA and TIVA groups and higher than usual mortality in the TIVA group: 3.3% (sevoflurane), 6.7% (desflurane) and 12.3% (TIVA) [42].

The first meta-analysis showing that the choice of anaesthetic regimen has an impact on patient outcome, including mortality, was published by Landoni’s group in 2007 [49]. They included 22 studies and a total of 1922 patients undergoing cardiac surgery (mainly CABG surgery with CPB) and reported a fourfold lower mortality rate [0.4% vs. 1.6% odds ratio (OR) 0.31(0.12–0.80); *p* = 0.02] with the use of either sevoflurane or desflurane compared to TIVA. A further Bayesian network meta-analysis performed by the same group, which included 38 cardiac surgery (63% CABG surgery with CPB) studies with 3966 patients observed that mortality, at the longest follow-up available, was doubled in patients receiving iv. anaesthesia compared with VA (2.6% vs. 1.3%), especially when sevoflurane or desflurane was used [50]. The statistical significance was reached exclusively when combining all volatile agents and the authors looked at the all-cause mortality, not that of cardiac origin only. Notably, approximately 40% of the weight of the effect was based on the trials published by De Hert and colleagues [20,23,42].

The mortality benefit of VA in cardiac surgery but not in non-cardiac surgery, has also been confirmed in a recent meta-analysis which included overall 68 RCTs (45 studies in cardiac surgery, 4890 patients) [51]. However, the results need to be interpreted with caution as the risk for bias was medium to high in most trials included in the analyses and none of these trials was powered for mortality as primary outcome.

Some studies looked specifically to different VA. A meta-analysis performed on isoflurane only showed a trend (*p* = 0.05) towards a reduction in mortality in a subgroup of high-quality studies comparing isoflurane to propofol in cardiac surgery [52]. Importantly, RIPC could also reduce mortality in patients receiving VA [53].

Recently, one large (868 patients) RCT performed in cardiac surgery confirmed the cardioprotective properties of sevoflurane in terms of cardiac biomarker release, reduced hospital LOS (10, IQR 9–11 vs. 14 days, IQR 10–16, *p* < 0.001) and mortality at 1-year follow up (17.8% vs. 24.8%, *p* <0.03) compared with the propofol-based TIVA group [40]. However, 7- and 30-day mortality were not reduced. Although the authors speculated on the role of propofol in increased mortality, analysis of the subgroups with and without any propofol administration throughout all anaesthesia time showed only a trend in the reduction of mortality in those not receiving propofol. Moreover, the study has been criticised for the high rate of lost to follow up and a substantial difference in 1-year mortalities, especially in the propofol group, as compared to other studies, which might influence the results [12,54]. These discussions indicate that long-term mortality might be influenced by factors other than anaesthesia and surgery.

In a further meta-analysis of 58 studies enrolling a total of 6105 participants in both on and off pump cardiac surgery, it was shown that sevoflurane reduces death within 180 to 365 days after surgery and inotropic and vasoconstrictor support compared to propofol for patients undergoing CABG surgery [55]. However, the methodological quality was difficult to assess as it was poorly reported in 35 included studies (3 or more domains were rated as unclear risk of bias).

In contrast, other authors did not find a mortality benefit or reduced incidence of MI, mechanical ventilation time and in-hospital LOS, but did find an improvement in CO, inotropic and vasoconstrictor drug use, ICU LOS, and incidence of atrial fibrillation [37].

Similarly, some recent large studies in cardiac surgery do not support the improved outcome by VA cardioprotective effect [56,57]. A multicentre RCT in patients undergoing high risk cardiac surgery has not observed any beneficial effect of anaesthesia with sevoflurane and desflurane compared with propofol based iv. anaesthesia, on the composite endpoint of prolonged ICU LOS, mortality (30 days and 1 year) or both [56]. In a large cohort study of cardiac surgery patients from three university hospitals in Denmark there were no differences in postoperative short- and long outcomes between VA and TIVA [57]. Interestingly, an old large retrospective study suggested a beneficial effect on survival with the use of sevoflurane as compared to propofol in low-risk cardiac surgery, without severe pre-operative ischaemia but not in patients with pre-operative unstable angina and/or recent MI, and thus already ‘preconditioned’ who might benefit from propofol’s anti-oxidant and anti-inflammatory properties [58].

The reasons of the contradictory results from previous and recent studies and meta-analyses may be related to the small sample size, lack of blinding in some studies, differences in anaesthesia protocols, surgery types and procedures, differences of patients’ conditions, such as diabetes, valvular defects, cardiomyopathy, ASA status, age and sex, and outcome definitions focused on surrogate markers of myocardial injury and organ dysfunction [28,59]. For example, VA and propofol were administered in any combination in the pre-, during and post-bypass period. Moreover, the most effective 1 MAC concentration at the time of reperfusion, was sometimes difficult to obtain in practice. Another major drawback is related to the fact that the role of opioids, ketamine and dexmedetomidine in cardioprotection were not considered in most studies comparing VA and TIVA [28]. Since the extent of cardioprotection may vary according to the protocol used, interpretation of the clinical relevance of cardioprotection in cardiac surgery remains a point of debate.

Despite these contradictory results, international web-based consensus conferences on mortality in cardiac surgery and in perioperative critical care medicine supported VA in cardiac surgery as a highly agreed non-surgical intervention contributing to increased postoperative survival [60,61].

In this context, favourable to VA in cardiac surgery, the results from the most recent large-scale RCT were unexpected [6]. The MYRIAD (Mortality in Cardiac Surgery Randomized Trial of Volatile Anesthetics) trial was a multicentre, single-blind, controlled trial that included patients scheduled to undergo elective isolated CABG in 36 centres from 13 countries. 5400 patients were randomly assigned to an intraoperative anaesthetic regimen that included a VA (desflurane, isoflurane, or sevoflurane) or TIVA. Whilst there was no strict inhalational anaesthesia protocol, the authors had recommended the use of cardioprotective strategies for patients receiving VA, including: (1) achieving a MAC of 1.0 for at least 30 min; (2) the wash out of VA at least 15 min prior to initiating CPB, and (3) performing at least three wash-in/wash-out periods, which were defined by administration of at least 0.5 MAC of the VA for 10 min interspersed by a wash-out period of 10 min or more. The primary outcome of the trial was death from any cause at 1-year. Secondary endpoints were 30-day mortality; 30-day death or non-fatal MI (composite endpoint); cardiac mortality at 30 day and at 1 year; incidence of hospital re-admission during the 1-year follow-up period and duration of ICU and hospital LOS. Intraoperative anaesthesia with a VA did not result in significant lowering the number of deaths at 1-year follow up as compared to TIVA [2.8% vs. 3.0%; relative risk (RR), 0.94; 95% CI 0.69 to 1.29; *p* = 0.71]. The same was found for death at 30 days (1.4% vs. 1.3%). No difference was observed for other secondary outcomes either. Moreover, there were no significant differences in the following aspects: adverse cerebral outcome (a composite of stroke, delirium, or postoperative cognitive impairment), AKI and requirement for RRT, surgical revision for bleeding, high-dose inotropic support and mechanical circulatory support. However, there was a reduction in hemodynamically significant MI in the VA group. No difference was found between on- or off-pump subgroups. Noticeably, the study was stopped for futility at its second interim analysis, reducing the power of the study and potentially leading to an underestimation of the treatment effect.

It is important to mention that only 64% of the study patients were operated on pump, with a mean duration of CPB of 79 min. In the absence of any severity scores mentioned, the short CPB duration and the mean preoperative left ventricle EF of 58% suggest inclusion of low-risk patients. Consequently, the incidence of LCOS was low. Knowing that potential effects of VA cardioprotection on outcome might be more easily detected in high-risk patients or in more complex procedures that are associated with much higher morbidity and mortality, large RCTs in higher-risk patients will be necessary to reassess the clinical evidence.

Other factors that might have contributed to the results include the pragmatic design of the study which left the attending anaesthesiologist free to choose the VA to use, the dose and duration of administration, the phases of surgery in which to administer it, and whether to use any iv. agent (e.g., opioids, propofol, midazolam) simultaneously. Propofol was co-administered during the induction of anaesthesia in 89% of VA patients and in 59% of them for maintenance, despite knowing that this may jeopardize the beneficial effect on myocardial preconditioning provided by VA [62]. Moreover, among patients in the VA group with available data, all three of the recommended strategies to enhance the cardioprotective effect of VA were used in only 10% of patients; 97% of patients in the VA group had at least one of the three strategies. The first strategy, as described above, was implemented in 92% of VA patients, the second strategy in 42% of patients and the third in 24% of VA patients, respectively. Only 478 patients in the VA group received VA during CPB. Knowing that the anaesthetic preconditioning effect may be dose-dependent and related to the timing and duration of administration of agents, and may be more pronounced with some agents over others, this variability of the protocol used might have influenced the results [2]. A fair comparison would have used the VA as the main anaesthetic agent throughout the whole procedure (i.e., for several hours), similar to the use in the TIVA group.

The above criticism points out that, although of large sample size, the MYRIAD RCT [6] is not convincing enough to end any further discussion on the topic of clinical relevance of anaesthetic cardioprotection.

A post hoc analysis of the MYRIAD trial, including 1586 patients using the same perioperative protocol at a single institution which represented the major contributor to the original study, found no significant difference in the primary outcome (mortality at 1 year 2.5% vs. 3.2%, *p* = 0.53) or in the rates of major complications, including MI, stroke, AKI, prolonged ventilation (>24 h), receipt of high-dose inotropic support (inotropic score >10), and need for mechanical circulatory support, duration of ICU LOS, hospitalization and hospital re-admission during follow-up between groups [63].

As expected, most conventional meta-analyses performed after the MYRIAD trial and including its large number of patients, do no longer support the benefit of VA regimen in cardiac surgery [64,65,66]. One meta-analysis included 89 RCTs comprising 14,387 CABG patients and showed that the use of VA during CABG was not associated with reduced risks of operative mortality, 1-year mortality, and postoperative safety outcomes when compared with TIVA [64]. The TSA showed that the results for in-hospital LOS, MI, arrhythmia, delirium, postoperative cognitive impairment, AKI, and the use of other mechanical circulatory support were conclusive, while the current evidence for operative and 1-year mortality as well as several other postoperative safety outcomes (heart failure, stroke, and the use of IABP) and ICU-LOS is insufficient and inconclusive. Thus, the authors conclude that the use of VA may not be superior to TIVA for CABG patients and further large RCTs are still needed to clarify this issue.

Another meta-analysis including 40 studies and over 10,000 patients with any type of cardiac surgery, found no statistically significant difference between patients receiving TIVA and VA in mortality, biomarkers of myocardial injury and duration of tracheal intubation but found a significant difference in favour of VA regarding the hospital and ICU LOS [66]. It is worth noting that the MYRIAD trial accounted for 57% of included patients. However, this meta-analysis has been criticised for including only 71% (618 of 868) of the patients from one trial [40] for reasons that are not explained [12].

In contrast, one recent meta-analysis including 42 studies and 8197 patients undergoing cardiac surgery with CPB (both CABG and valve or complex surgery) found that 1-year mortality was significantly lower in patients who received VA for maintenance of anaesthesia as compared to propofol [5.5% vs. 6.8%; OR 0.76 (95% CI, 0.60 to 0.96); *p* = 0.023] [67]. The incidence of perioperative MI, need for inotropic medication and levels of cTn release were lower and the postoperative cardiac indexes were higher in the VA, whereas short mortality was similar in the VA and propofol groups. Due to the lack of homogeneity of trials the authors suggest the need of new trials to clarify the effect of VA on short- and long-term mortality. Notably, this meta-analysis included studies which used VA for the entire intervention excluding those on pre- or postconditioning only. However, there were studies in which iv. anaesthetics were used for some period in the VA group, which may have attenuated the favourable effect. Noteworthy, this meta-analysis was criticised for excluding patients who underwent OPCAB (almost 2000 patients), which also meant that only a sub-cohort of those included in the MYRIAD trial were analysed, which was considered to result in an unacceptable risk of bias [12].

Intriguingly, the 2 meta-analyses discussed above [66,67] included slightly different studies, which may explain the contradictory conclusions [12]. Moreover, neither one included an old RCT by Slogoff and Keats [68], which showed that outcomes of patients undergoing cardiac surgery were similar when VA were compared with a TIVA (benzodiazepine and sufentanil) and pointed out the role of the anaesthesia management in the rate of perioperative complications.

In contrast to all of the above studies, a large South Korean retrospective database review comparing TIVA with volatile maintenance on adverse outcomes after primary CABG surgery found a 3% absolute risk reduction in mortality that persisted up to 3 years after surgery favouring TIVA, suggesting for the first time that VA are associated with increased mortality in the long term [69]. However, the data should be interpreted with caution as the drawback of retrospective studies can be only partially compensated for by propensity score analysis. Moreover, as highlighted by the authors, geographical differences may contribute to the results, as it was shown that Asian patients are more sensitive to propofol [70].

A summary of the currently published relevant studies on the role of anaesthetic agents on mortality in cardiac surgery is available in Table 1. New data from ongoing RCTs, such as the VIRS trial (clinical trial number: ChiCTR-IOR-17013578), a large, multi-centre RCT comparing VA and TIVA in cardiac surgery, which included 3100 patients, will hopefully bring more clarity on this topic.

### 3.3. Perioperative Myocardial Infarction

Perioperative MI in patients undergoing CABG surgery has a wide range of presentations, from clinically silent to hemodynamically significant, including LCOS, difficult weaning from CPB, concomitant vasoplegia and vasodilatory shock, AKI and stroke, which increase the risk of adverse short and long-term outcomes as compared with asymptomatic patients [71]. By attenuating apoptosis and necrosis, VA may reduce the infarct size and myocardial dysfunction after IR injury [15,19]. Significantly lower incidence of MI was found in the VA groups compared to TIVA in a couple of meta-analyses of RCTs, mostly in CABG surgery [49,50,55].

A recent post hoc analysis of the MYRIAD trial performed in CABG patients [6], having as primary outcome hemodynamically relevant MI (MI requiring high-dose inotropic support or prolonged ICU LOS) occurring within 48 h from surgery and as a secondary outcome 1-year death due to cardiac causes, found that patients receiving VA intraoperatively had a lower incidence of MI with haemodynamic complications when compared to TIVA in the per-protocol analysis (0.6% vs. 1.1%, *p* = 0.038) and as-treated analysis (0.6% vs. 1.1%, *p* = 0.039), but not in the intention-to-treat analysis (0.6% vs. 1%, *p* = 0.1) [72]. Overall, deaths due to cardiac causes (cardiogenic shock and arrhythmias) were lower in the VA group (0.9% vs. 1.5%, *p* = 0.03). Patients who developed MI with hemodynamic complications had a 1-year mortality rate of 25%, which is 10 times higher as compared with patients who did not develop this complication. However, these findings should be considered hypothesis-generating only as MI was a secondary outcome in the original trial, which was not powered to detect the differences in perioperative MI with hemodynamic complications [6]. Importantly, cTn level was not different between groups in the original study, but it was not a study parameter, and the release has been monitored variably in different centres. The very low incidence of the primary outcome and inadequate sample to detect differences in perioperative MI make the results of the post-hoc analysis difficult to generalize.

### 3.4. Neurologic Complications

Stroke and encephalopathy are two main neurologic complications after cardiac surgery, associated with high rate and increased morbidity and mortality [73]. Encephalopathy includes confusion, delirium, seizures, coma, and prolonged alteration in mental status, combativeness, agitation, and postoperative cognitive function decline within 3 months after surgery, which is elusively named postoperatively cognitive dysfunction (POCD) [73]. POCD can affect as high as 50% of patients after cardiac surgery and is associated with slower recovery, increased hospital admissions, reduced rehabilitation adherence, early retirement from work and increased risk of Alzheimer disease [74].

Currently, there are no confirmed interventions for protecting the brain in patients undergoing cardiac surgery with CPB and the role of anaesthetic regimen in neuroprotection in cardiac surgery is not clear. VA can ideally provide neuroprotective effects through similar pathways as in cardioprotection [75]. Moreover, VA increases the brain tolerance to hypoxia by reducing the cerebral metabolic rate (CMRO2) and decreases the glutamate-induced excitation, which is crucial in neuronal injury [76]. Similarly, iv. anaesthetic agents also have properties which may increase tolerance to ischaemia. They reduce the CMRO2, supress neurotransmission, decrease electrophysiologic brain activity and preserve the energy balance during transient interruption of substrate delivery [76]. Moreover, propofol protects neurons against oxidative stress and can supress apoptosis and inflammation, and decrease both the CMRO2 and cerebral blood flow (CBF) in a dose-dependent way with preservation of cerebral autoregulation [31]. Despite these potential favourable effects, the clinical studies on the effects of VA or TIVA on the rate of postoperative neurological complications in non-cardiac surgery suggest that patient and surgical variables may be more important than the anaesthetic technique [77]. Moreover, it seems that the POCD rate is similar after on- and off-pump surgery [78]. The first systematic review and meta-analysis including 13 on-pump cardiac surgery RCTs and a total of 549 patients comparing the neuroprotective effects of inhalational anaesthesia (isoflurane, sevoflurane and desflurane) to TIVA (propofol, thiopental, midazolam, and ketamine) found significantly lower post-CPB and postoperative blood level of S100B, an early marker of brain injury and neurologic dysfunction, in the VA group, suggesting the potential superiority of VA for neuroprotection over TIVA [79]. Among secondary outcome variables, mini-mental state examination scores were significantly higher in the VA group than in the TIVA group 24 h after surgery, suggesting that VA is better than TIVA in terms of protection of cognitive function. No significant difference was found in arteriovenous oxygen content difference, cerebral oxygen extraction ratio and jugular bulb venous oxygen saturation, which were assessed at cooling and rewarming during CPB.

Another study compared the combination of propofol and fentanyl to sevoflurane for maintenance of anaesthesia in off-pump CABG [80]. The reduction in specific scores was significantly lower in the sevoflurane group compared to the propofol/fentanyl group. Additionally, there was a significant larger increase in inflammatory markers (CRP, TNF-a, IL-6) in the propofol/fentanyl group compared to the sevoflurane group following surgery, suggesting better anti-inflammatory effects of sevoflurane, compared to propofol/fentanyl. The lower levels of intraoperative malondialdehyde within the sevoflurane group supported the role of VA in scavenging ROS and attenuation of oxidative stress.

In contrast, the MYRIAD trial did not find differences between VA and TIVA in the incidence of stroke, delirium, or postoperative cognitive impairment [6]. Unfortunately, this study did not use specific tests for assessing these complications.

Similarly, in a recent systematic review and meta-analysis of 89 studies comparing VA and TIVA in CABG surgery patients, from the eight studies within the meta-analysis reporting on POCD, there was no significant difference between VA and TIVA [64]. It should be noted that quality of the studies included for this outcome were rated as low by the authors.

Intriguingly, a single-site sub-study of a multi-centre RCT on awareness in high risk surgery that included 310 cardiothoracic surgery patients randomly assigned to receive either bispectral index (BIS)-guided or end-tidal agent concentration-guided inhalational anaesthesia showed an increased incidence of postoperative delirium in patients who received lower doses of VA [81].

Others strongly support the use of propofol over VA to reduce the incidence of POCD following cardiac surgery. One study comparing the effect of intraoperative sevoflurane (given to maintain end-expiratory and end-effluent concentrations of 1–3%) vs. propofol (infused to reach a serum concentration of 0.5–2.0 ug/kg/min with a BIS index of 40–55) on POCD following on-pump cardiac surgery found a significant lower incidence at 12 (9.09% vs. 21.82%) and 24 (10.91% vs. 25.45%) hours postoperatively in the propofol group compared to the sevoflurane group [82].

The effects of the anaesthetic regimen on postoperative delirium in transcatheter aortic valve replacement (TAVR) patients are also debated. In a retrospective study, patients who had a TIVA maintenance technique for TAVR experienced significantly less delirium as compared with those on VA [83]. On the other hand, those operated under monitored anaesthesia care (MAC) had lower in-hospital LOS than those under general anaesthesia (iv. or balanced regimen) and general anaesthesia was found to be a risk factor for delirium [84]. In contrast, in RCTs there was no difference in 30 day- and 1 year outcome between MAC and general anaesthesia [85,86].

Based on the existing evidence, the effects of the anaesthetic regimen on postoperative neurological complications, if any, remain controversial. Moreover, most clinical trials used surrogate markers of neurologic injury. Overall, the present evidence suggests that surgery, perfusion and patient-related risk factors have a greater impact on cognitive functions than anaesthesia-related risk factors [87]. Ongoing RCTs studying the impact of anaesthetic technique on POCD complications, such as postoperative delirium (clinical trial number: ChiCTR1900021355) might bring more clarity into this topic.

### 3.5. Acute Kidney Injury

AKI is another frequent complication of cardiac surgery which is caused by many factors including IR damage, CPB and inflammation [88]. As IPC has been demonstrated in organs other than the heart, anaesthetic preconditioning might also have the potential for renal protection in cardiac surgery. Recent studies suggest that modern VA induce potent anti-inflammatory, antinecrotic, and antiapoptotic effects that protect against ischaemic AKI [89]. However, the mechanism is likely different from cardiac IR injury protection and need the presence of VA during renal ischaemia to provide protection.

A meta-analysis including a total of 1600 cardiac surgery patients from 10 RCTs found a lower incidence of postoperative AKI (RR 0.65, 95% CI 0.43–0.97; *p* = 0.04) and a reduced rate of prolonged ICU and hospital LOS in patients who received VA compared with TIVA [90]. VA significantly improved the change from baseline in the level of serum creatinine, although there were no differences in the absolute serum creatinine levels and the rate of RRT between the groups. Unfortunately, the definition of postoperative AKI was not uniform. Noteworthy, there was a borderline significantly lower level of cystatin C in the VA group, which is a more accurate biomarker of renal dysfunction. Importantly, a significant reduction in the need for intraoperative and postoperative inotrope therapy was found in the VA group, which suggested that VA administration was associated with better hemodynamic stability.

In contrast, a further RCT performed in 112 patients undergoing heart valve surgery reported that propofol-based anaesthesia reduced the incidence of AKI by more than a third when compared with sevoflurane [91]. The severity of AKI in the propofol group, when it did occur, was also reduced. Sevoflurane significantly increased the levels of cystatin C and other inflammatory biomarkers, thus suggesting a better protective role of propofol, which might have a better ability to attenuate perioperative increases in pro-inflammatory mediators. The discrepancy to previous studies was explained by the use of an adequate propofol target concentration, known to exert cardioprotection. Propofol dose, baseline renal function and definition of AKI are potential contributing factors to the rate of AKI [92].

Furthermore, a recent systematic review and meta-analysis evaluating the effects of propofol vs. VA on mortality and major clinical events in cardiac surgery patients revealed that the incidence of AKI and RRT was similar between the two anaesthesia regimens, and concluded that VA offered no renal protection, probably due to different studies included [67].

Notably, two meta-analyses evaluating the effects of RIPC showed a reduction in AKI in the subgroup of studies in which propofol was not used, suggesting that propofol may interact with the protective effects of RIPC [53,93]. A recent large meta-analysis confirmed that RIPC significantly reduced the incidence of postoperative AKI (22% vs. 24.4%), especially in volatile only anaesthesia, in non–high-risk patients, and with the use of Acute Kidney Injury Network (AKIN) or Kidney Disease Improving Global Outcome (KDIGO) criteria for AKI diagnosis, suggesting that the benefits of RIPC in cardiac surgery may depend on both the choice of anaesthetic agent and the patients’ risk profile [94]. However, another meta-analysis which supports RIPC as an effective strategy to prevent AKI after cardiac surgery did not find a significant impact of anaesthesia agents on the efficacy of RIPC on postoperative AKI in meta-regression and subgroup analysis [95].

Interestingly, renal function was better preserved in patients anaesthetized with a combination of sevoflurane and propofol, supporting an enhanced protective effect on renal function by both sevoflurane and propofol [96].

### 3.6. Pulmonary Complications

The CPB-induced systemic inflammatory response and lung IR injury were associated with pulmonary dysfunction [97], which can result in airway constriction, atelectasis, hypoxemia, elevated right heart afterload, and reduced systemic venous return [98].

A potential role of the anaesthetic regimen on the rate of postoperative pulmonary complications (PPCs) in cardiac surgery has been suggested [2]. Proposed mechanisms for VA lung protection include anti-inflammatory, antioxidant, and anti-apoptotic effects on endothelial cells, immunocompetent cells, and epithelial cells by inhibiting nuclear factor kappa B (NF-kB), mitogen-activated protein kinase (MAPK), and protein kinase C (PKC) signalling, as well as by inhibiting overexpression of inducible nitric oxide synthase (iNOS) and maintaining expression of endothelial nitric oxide synthase (eNOS) [99]. These lung protective effects of VA might be triggered by the small amounts of ROS induced by VA. VA also exert a lung protective effect on the glycocalyx present on the surface of pulmonary endothelium and on the tight junctions between alveolar epithelial and endothelial cells [99].

Clinical studies comparing the effects of VA and propofol-based TIVA on PPCs have provided conflicting results. The results in thoracic surgery with one lung ventilation (OLV), which are relevant for mini-invasive cardiac surgery with lateral thoracotomy, showed fewer PPCs and lower levels of inflammatory biomarkers with sevoflurane vs. propofol [100], and improved lung collapse, significantly shortened video-assisted lobectomy operation time and fewer PPCs in the desflurane group compared to propofol [101]. In contrast, another clinical trial failed to prove the superiority of VA over propofol-based TIVA [102].

On the other hand, it seems that propofol reduces the intraoperative shunt and maintains better oxygenation during OLV as compared to VA [103]. In contrast, TIVA did not better preserve V/Q matching in patients with normal lungs undergoing anaesthesia with controlled ventilation compared with sevoflurane [104].

In cardiac surgery, besides protective lung effects, sevoflurane was found to alleviate CPB-induced bronchoconstriction with subsequent development of atelectasis and intrapulmonary shunt, a beneficial effect particularly important in patients with severe

CPB-induced lung function deterioration [98]. However, the benefit of VA in reducing PPCs in cardiac surgery is controversial.

A meta-analysis of 68 RCTs that compared VA with TIVA in different types of surgeries, found a significant reduced risk of PPCs in patients undergoing cardiac surgery (1507 patients from 12 trials) but not in those undergoing non-cardiac surgery [51]. However, this meta-analysis included heterogeneous studies that had small population sample sizes and various definitions of outcomes making the findings difficult to generalize and increasing the risk of bias.

A recent meta-analysis of 36 studies on patients with CABG, including MYRIAD trial, showed that intraoperative use of VA compared to TIVA might shorten the mechanical ventilation time and the ICU LOS suggesting a protective effect of VA on pulmonary tissue [65]. This is consistent with a previous meta-analysis on sedative selection in ventilated critical care patients [105], as well as a retrospective review on the use of VA as sedatives for ARDS [106], where inhaled anaesthetics reduced mechanical ventilation time.

In contrast, in one RCT which included 524 patients undergoing cardiac surgery with CPB, mainly isolated valvular replacements, VA (sevoflurane or desflurane) administered during the entire surgical procedure, compared with propofol-based TIVA had not reduced PPCs (both the occurrence and severity) within the first 7 days after surgery [107].

## 4. What about Other Types of Cardiac Surgery?

Among patients undergoing heart valve surgery under CPB, the use of VA compared with TIVA failed to demonstrate superiority for survival and major postoperative complications [108]. Although sevoflurane anaesthesia produced more prominent myocardial protection, attenuated the inflammatory response, and had a higher ratio of automatic heart beat recovery compared to propofol anaesthesia, which resulted in shorter ICU and in-hospital LOS, the authors concluded that evidence was insufficient to draw firm conclusions due to the limited sample size [108].

Another study found that the intramyocardial delivery of sevoflurane produces a stronger attenuation of the systemic inflammatory response after CPB for mitral valve surgery, without reducing postoperative markers of myocardial cell damage, compared with systemic sevoflurane or propofol groups [109]. This may suggest that the relation between systemic inflammation and myocardial protection may be more complex than commonly thought.

The first meta-analysis of RCTs comparing VA and TIVA in heart valve surgery, including 13 studies and 962 patients, found no difference in terms of mortality (4.8% vs. 5.3%) and similar effects on cardioprotection (postoperative cTn release, incidence of arrhythmia and MI), renal protection (AKI incidence), PPCs, neurological events, postoperative bleeding, ventilation time, in-hospital and ICU LOS [110]. However, the TSA for postoperative peak cTn release, AKI, postoperative arrhythmia, ventilation time and ICU LOS revealed further investigation is warranted in valve surgery [110].

OPCAB surgery is also associated with myocardial ischaemia, resulting from transient coronary occlusion to facilitate graft anastomoses, decreases in coronary perfusion pressure occurring when the position of the heart is changed, and use of vasoactive medications to treat hypotension, which may cause increases in myocardial oxygen demand in the presence of flow-limiting coronary stenoses [12]. However, the IR injury seems to be less prominent compared to on-pump CABG surgery and therefore anaesthetics might have a smaller effect. Consequently, OPCAB surgery studies have drawn conflicting conclusions, showing no benefit of VA on the incidence of in-hospital and 1-year major adverse events [111] but lower incidence of postoperative arrhythmias [111], and lower myocardial injury [45] as compared to propofol. The MYRIAD trial also demonstrated that the use of VA was not associated with reductions in short and long term mortality nor did it offer any other distinct outcome advantage vs. TIVA, independent of whether CPB was used or not in elective CABG [6].

In contrast, one study that also used sedation with sevoflurane in the postoperative period concluded that this might have enhanced the cardioprotective effect of sevoflurane compared to propofol [45]. This was confirmed in a further RCT which showed less myocardial and renal injury in patients receiving sevoflurane both intraoperatively and postoperatively compared to propofol or intraoperative only sevoflurane groups [46].

## 5. Conclusions

In conclusion, given the conflicting evidence presented in this review, there remains considerable scientific equipoise regarding the best anaesthetic regimen for cardiac surgery with CPB. Presently, it is difficult to conclude whether one anaesthetic approach is superior to the other in terms of patient outcome and there is no strong recommendation on the use of a specific regimen. Whether anaesthetic preconditioning truly contributes to the anti-ischemic effects of VA and these effects are translated into improved outcome in patients undergoing cardiac surgery at risk for perioperative myocardial ischemia remains to be definitively established. Factors such as nonuniform extent of the ischemic insult, the type of surgery, time of aortic cross clamping and type of cardioplegia, the presence of comorbidities, the effects of concurrent medication, different anaesthesia protocols (timing, dosing, type of agent) and definitions of outcome may influence the results of different studies. Large RCTs including high-risk patients, homogeneous for surgical and anaesthesia protocols, are needed to assess the impact of the anaesthetics.

On the other hand, the IR injury in cardiac surgery is too complex to be targeted by one single intervention, such as the choice of the anaesthetic regimen. Most probably, the perioperative outcome of cardiac surgery patients depends more on how the anaesthesiologists use the available tools, anaesthetic agents, adjuvants, and vasoactive drugs to control the homeostasis of these patients and manage temperature, haemoglobin levels, haemostasis, cardiovascular changes, glycaemic control, protective ventilation, and other factors which may also affect the outcome. The selection of the anaesthetic regimen should also consider the technical and pharmacokinetic challenges focused on each patient’s demands. The skill and dedication of the anaesthesiologist are probably much more important than which drugs are being used. The paradigm may change in the future by the use of perioperative genomics and pharmacogenetics which will provide a more personalised anaesthesia approach.

## Figures and Tables

**Table 1:** Selected studies comparing mortality between VA and TIVA.

First Author, Year	Type of Study	Type of Surgery	Number of Patients	Volatile Agent(s)	IV Agent(s)	Findings (VA vs. TIVA)
Landoni, 2007 [49]	Meta- analysis of RCTs	Cardiac surgery	1922	Sevoflurane, desflurane	Propofol and others	In-hospital mortality was 0.4% vs. 1.6%
De Hert, 2009 [42]	RCT	On-pump CABG	414	Sevoflurane, desflurane	Unspecified	Mortality was 12.3% in TIVA group, 3.3% in sevoflurane group and 6.7% in desflurane group
Landoni, 2013 [50]	Bayesian network meta- analysis	Cardiac surgery	3996	Sevoflurane, isoflurane, desflurane	Propofol and others	Mortality was 1.3% vs. 2.6% at longest available follow up Sevoflurane and desflurane, but not isoflurane, were associated with reduction
Landoni, 2014 [56]	RCT	High-risk cardiac surgery	200	Sevoflurane	Propofol	No difference in mortality
Li, 2015 [37]	Meta- analysis of RCTs	Cardiac surgery	1646	Sevoflurane	Propofol	No significant difference in mortality (OR 0.73, 95% CI 0.14–3.78, *p* = 0.71)
Uhlig, 2016 [51]	Meta- analysis of RCTs	Cardiac and non-cardiac surgery	4840/7104 patients cardiac	Sevoflurane, isoflurane, desflurane	Propofol and others	In cardiac surgery, VA was associated with reduced overall mortality (OR = 0.55; 95% CI, 0.35 to 0.85; *p* = 0.007)
Lickhvantsev, 2016 [40]	RCT	Elective CABG	868	Sevoflurane	Propofol	Mortality at 1 year was 17.8% vs. 24.8%, *p* = 0.03. 7-day and one-month mortality were not different.
El Dib, 2017 [55]	Meta- analysis of RCTs	On-pump and off-pump CABG	6105	Sevoflurane, Isoflurane, Desflurane, Enflurane	Propofol	Sevoflurane was associated with a reduction in death within 180 to 365 days of on-pump surgery (RR 2.11, 95% CI 1.53–2.9, *p* < 0.00001, I^2^ = 0%). Other VA agents did not show benefit.
Landoni, 2019 [6]	RCT	Elective on-pump and off-pump CABG	5400	Sevoflurane, Isoflurane, Desflurane,	Propofol, midazolam and others	All-cause mortality at 1 year was 2.8% vs. 3%, RR 0.94, 95% CI 0.69–1.29, *p* = 0.71. The trial was stopped for futility
Jiao, 2020 [64]	Meta-analysis of RCTs and TSA	CABG	14,387	Any	Any	No significant differences in operative mortality (RR = 0.92, 95% CI 0.68–1.24, *p* = 0.59, I^2^ = 0%), or 1-year mortality. TSA found evidence to be insufficient and inconclusive.
Zhang, 2020 [65]	Meta- analysis of RCTs	CABG	10308	Any	Any	30-day mortality was 1.4% vs. 1.3%, RR = 1.11, 95% CI 0.7–1.74, *p* = 0.66, I^2^ = 0%). 1 year mortality was not different either.
Bonanni 2020 [67]	Meta- analysis of RCTs	On-pump CABG	8197	Any	Propofol	1-year mortality was 5.5% vs. 6.8%, OR 0.76 (95% CI 0.60–0.96), *p* = 0.023. Short term mortality was 1.63 vs. 1.65%, OR 1.04, 95% CI 0.73–1.49, *p* = 0.820.
Beverstock, 2021 [66]	Meta- analysis of RCTs	Cardiac surgery	10,886	Sevoflurane, desflurane, isoflurane	Any	No difference in one-year mortality (*n* = 6440, OR 1.22, 95% CI 0.97–1.54, *p* = 0.09, Z = 1.67, I^2^ = 0%.

Abbreviations: RCT—randomized controlled trial, CABG—coronary artery by-pass grafting, VA—volatile anaesthesia, TIVA—total intravenous anaesthesia, OR—odds ratio, CI—confidence interval, RR—relative risk, TSA—trial sequential analysis.

## Data Availability

Not applicable.
